# A molecular phylogeny of the Cephinae (Hymenoptera, Cephidae) based on mtDNA COI gene: a test of traditional classification

**DOI:** 10.3897/zookeys.130.1466

**Published:** 2011-09-24

**Authors:** Mahir Budak, E. Mahir Korkmaz, Hasan H. Basibuyuk

**Affiliations:** 1 Department of Biology, Faculty of Science, Cumhuriyet University, 58140-Sivas, Turkey; 2 Department of Molecular Biology and Genetics, Faculty of Science, Cumhuriyet University, 58140-Sivas, Turkey

**Keywords:** Hymenoptera, phylogeny, Cephidae, COI, host shift

## Abstract

Cephinae is traditionally divided into three tribes and about 24 genera based on morphology and host utilization. There has been no study testing the monophyly of taxa under a strict phylogenetic criterion. A molecular phylogeny of Cephinae based on a total of 68 sequences of mtDNA COI gene, representing seven genera of Cephinae, is reconstructed to test the traditional limits and relationships of taxa. Monophyly of the traditional tribes is not supported. Monophyly of the genera are largely supported except for *Pachycephus*. A few host shift events are suggested based on phylogenetic relationships among taxa. These results indicate that a more robust phylogeny is required for a more plausible conclusion. We also report two species of *Cephus* for the first time from Turkey.

## Introduction

The Cephidae is a small family of Hymenoptera with a thin integument, usually black or dark colored and commonly with narrow yellow bands on the abdomen. It comprises approximately 160 species in three subfamilies and about 24 genera and is primarily Holarctic (Benson 1935, 1946; Smith and Schmidt 2009; Taeger et al. 2010). Two of the subfamilies, Athetocephinae and Australcephinae are represented by only four species and are restricted to Madagascar and the Australian Region (Benson 1935, 1946; Smith and Shinohara 2002a; Smith and Schmidt 2009). The majority of species are included in the Holarctic subfamily Cephinae. Although several faunal treatments (Ries 1937; Middlekauff 1969; Zhelochovtsev and Zinovjev 1988; Calmasur and Ozbek 2010; Korkmaz et al. 2010) and a single world review (Muche 1981) have been published, and a number of cephid species have been described in recent years (Smith and Solomon 1989; Smith 1997; Smith and Shinohara 2002a, b; Smith and Schiff 2005; Wei 2007; Smith and Schmidt 2009; Wei and Smith 2010), their phylogenetic relationships have not been investigated.

Cephidae can be easily identified since they are morphologically intermediate between the hymenopteran suborders Symphyta and Aculeata. Because of several apocritan-like characters, such as a weak constriction between the first and second abdominal segments, the lack of cenchri and the rough area on fore wing, and the form of male genitalia, they were once considered as a likely sister group of Apocrita (Königsmann 1977). However, considerable evidence from both morphological and molecular data strongly support a sister group relationship between Orussidae and Apocrita, and the Cephoidea, containing the only family Cephidae, appears as to share a last common ancestor with a lineage leading to the Siricoidea (e.g., Rasnitsyn 1980, 1988; Basibuyuk and Quicke 1995; Vilhelmsen 1997, 2001; Ronquist et al. 1999; Schulmeister et al. 2002; Sharkey 2007).

Ries (1937) suggested that *Janus* is the most primitive genus of Cephinae based on its filiform and many segmented antennae and tarsal claws. Benson (1946) divided the Cephinae into three tribes, Cephini, Hartigiini and Pachycephini based both on morphology and host utilization. The larvae of Cephini bore in the stems of Poaceae, those of Pachycephini live in the stems of Papaveraceae and Poaceae, and those of Hartigiini bore in the twigs of Rosaceae or other arborescent plant families (Benson 1946; Middlekauff 1969). Numerous morphological characters and color patterns that traditionally have been used for separation of the taxa within the family are claimed to be either variable or display phenotypic plasticity (Ries 1937; Benson 1946, 1968; Korkmaz et al. 2010). Current classification is mainly based on morphology and host usage and therefore necessitates a close examination under the phylogenetic approaches.

Phylogenetic studies of taxa that exhibit adaptive phenotypic variation provide valuable insights into the evolutionary forces driving the origins of diversification (Zhang et al. 2008). Research on phytophagous insects has confirmed that adaptation and specialization to different plant species are central to generating diversification at all hierarchical levels (Mopper and Strauss 1998; Berlocher and Feder 2002; Funk et al. 2002; Nosil et al. 2002; Eubanks et al. 2003; Lozier et al. 2007). However, host specialization in the Cephinae, as observed in most phytophagous insect groups, might have led to evolutionary shift between higher taxonomic groups. Host shift probably has taken place many times at different periods, and therefore the classification based on host use may not reflect the true phylogenetic relationship within the Cephinae.

Here, we selected the mitochondrial cytochrome oxidase subunit I (COI) gene to reconstruct the phylogenetic relationships of the Cephinae and identify systematic position of its tribes and genera by applying phylogenetic inference methods. The selected COI gene region is informative for estimating relationships at both intra- and interspecies level due to possession of both completely conserved and variable regions and having a heterogeneous evolutionary rate across the gene (Lunt et al. 1996; Dowton and Austin 1997; Caterino et al. 2000; Roe and Sperling 2007; Bacci et al. 2009) This region is also utilized as DNA–based bio-identification system for animals at the global level (Hebert et al. 2003). *Cephus parvus* (Dovnar-Zapolskij, 1931) and *Cephus runcator* Konow, 1896, are recorded for the first time from Turkey.

## Material and methods

Sixty-eight specimens representing three tribes and seven genera of the subfamily Cephinae were collected from localities presented in Table 1. All specimens are deposited in the Entomological Collection of Cumhuriyet University, Sivas (ECCUS). A specimen of *Arge* sp. (Argidae) was included as an outgroup in the phylogenetic analyses. Several keys were used to identify the specimens (Benson 1946, 1951, 1968; Muche 1981; Zhelochovtsev and Zinovjev 1988). The taxa names, the voucher specimens, and GenBank accession numbers are presented in Table 1.

**Table 1.** List of taxa and voucher specimens used for sequencing.

Genus	Species	Voucher no.	GenBank accession no.	Location	Col. date
Arge	Arge sp.	ECCUS 201	JF901916		
*Calameuta*					
	*Calameuta filiformis* (Eversmann, 1847)	ECCUS 210	JF901849	İçel	12.04.2009
	*Calameuta filiformis*	ECCUS 211	JF901850	Sivas	04.06.2009
	*Calameuta haemorrhoidalis* (Fabricius, 1781)	ECCUS 212	JF901852	Kütahya	20.05.2009
	*Calameuta haemorrhoidalis*	ECCUS 213	JF901853	Isparta	17.05.2009
	*Calameuta haemorrhoidalis*	ECCUS 214	JF901855	Kocaeli	04.05.2010
	*Calameuta haemorrhoidalis*	ECCUS 215	JF901856	Kocaeli	04.05.2010
	*Calameuta haemorrhoidalis*	ECCUS 216	JF901857	Bayburt	05.06.2010
	*Calameuta haemorrhoidalis*	ECCUS 217	JF901858	Uşak	19.05.2009
	*Calameuta haemorrhoidalis*	ECCUS 218	JF901859	Isparta	17.05.2009
	*Calameuta idolon* (Rossi, 1794)	ECCUS 219	JF901851	Konya	17.05.2009
	*Calameuta pallipes* (Klug, 1803)	ECCUS 220	JF901854	Sivas	13.05.2010
	*Calameuta pallipes*	ECCUS 221	JF901860	Hakkari	11.06.2003
	*Calameuta pygmaea* (Poda, 1761)	ECCUS 222	JF901848	Hatay	09.04.2009
	*Calameuta* sp.	ECCUS 223	JF901861	Sivas	17.06.2007
*Cephus*					
	*Cephus brachycercus* Thomson, 1871	ECCUS 230	JF901871	İstanbul	08.05.2010
	*Cephus brachycercus*	ECCUS 231	JF901872	Sivas	10.05.2010
	*Cephus fumipennis* Eversmann, 1847	ECCUS 232	JF901873	Ardahan	07.06.2010
	*Cephus nigrinus* Thomson, 1871	ECCUS 233	JF901874	İstanbul	08.05.2010
	*Cephus parvus* (Dovnar-Zapolskij, 1931)	ECCUS 234	JF901875	Sivas	17.05.2010
	*Cephus parvus*	ECCUS 235	JF901876	Sivas	26.05.2010
	*Cephus pulcher* Tischbein, 1852	ECCUS 236	JF901877	Erzurum	06.06.2010
	*Cephus pygmeus* (Linné, 1767)	ECCUS 237	JF901911	Denizli	18.05.2009
	*Cephus pygmeus*	ECCUS 238	JF901912	Hatay	09.04.2009
	*Cephus pygmeus*	ECCUS 239	JF901913	Hatay	09.04.2009
	*Cephus pygmeus*	ECCUS 240	JF901914	Bayburt	07.06.2008
	*Cephus pygmeus*	ECCUS 241	JF901915	Bayburt	07.06.2008
	*Cephus rjabovi* Dovnar-Zapolskij, 1926	ECCUS 242	JF901878	Kırıkkale	20.06.2009
	*Cephus rjabovi*	ECCUS 243	JF901879	Kırıkkale	20.06.2009
	*Cephus runcator* Konow, 1896	ECCUS 244	JF901880	Edirne	07.05.2010
	*Cephus runcator*	ECCUS 245	JF901881	Edirne	07.05.2010
	*Cephus sareptanus* Dovnar-Zapolskij, 1928	ECCUS 246	JF901882	Erzurum	06.06.2010
	*Cephus sareptanus*	ECCUS 247	JF901883	Erzurum	06.06.2010
	*Cephus* sp.	ECCUS 248	JF901884	Bilecik	05.05.2010
	*Cephus* sp.	ECCUS 249	JF901885	Bilecik	05.05.2010
	*Cephus* sp.	ECCUS 250	JF901886	Çanakkale	06.05.2010
	*Cephus* sp.	ECCUS 251	JF901887	Amasya	02.05.2010
	*Cephus* sp.	ECCUS 252	JF901888	Amasya	02.05.2010
	*Cephus* sp.	ECCUS 253	JF901889	Tekirdağ	08.05.2010
	*Cephus* sp.	ECCUS 254	JF901890	Sivas	18.05.2010
	*Cephus* sp.	ECCUS 255	JF901891	Erzurum	06.06.2010
	*Cephus* sp.	ECCUS 256	JF901892	Kars	07.06.2010
	*Cephus* sp.	ECCUS 257	JF901893	Kars	07.06.2010
	*Cephus* sp.	ECCUS 258	JF901894	Bolu	04.05.2010
*Trachelus*					
	*Trachelus iudaicus* (Konow, 1907)	ECCUS 260	JF901865	Bayburt	05.06.2010
	*Trachelus iudaicus*	ECCUS 261	JF901866	Bayburt	05.06.2010
	*Trachelus libanensis* (André, 1881)	ECCUS 262	JF901867	İçel	13.04.2009
	*Trachelus libanensis*	ECCUS 263	JF901868	İçel	13.04.2009
	*Trachelus* sp.	ECCUS 264	JF901862	Sivas	12.06.2010
	*Trachelus* sp.	ECCUS 265	JF901863	Sivas	30.05.2010
	*Trachelus tabidus* (Fabricius, 1775)	ECCUS 266	JF901869	İçel	12.04.2009
	*Trachelus tabidus*	ECCUS 267	JF901870	Çanakkale	06.05.2010
	*Trachelus troglodyta* (Fabricius, 1787)	ECCUS 268	JF901864	Zonguldak	03.05.2010
*Hartigia*					
	*Hartigia linearis* (Schrank, 1781)	ECCUS 270	JF901896	Ardahan	07.06.2010
	*Hartigia linearis*	ECCUS 271	JF901897	Kırşehir	03.06.2003
	*Hartigia linearis*	ECCUS 272	JF901898	Kırşehir	03.06.2003
	*Hartigia nigra* (M. Harris, 1779)	ECCUS 273	JF901899	Konya	17.05.2009
	*Hartigia* sp.	ECCUS 274	JF901900	Sivas	17.05.2010
	*Hartigia* sp.	ECCUS 275	JF901901	Sivas	13.05.2010
	*Hartigia xanthostoma* (Eversmann, 1847)	ECCUS 276	JF901902	Zonguldak	03.05.2010
	*Hartigia xanthostoma*	ECCUS 277	JF901903	Zonguldak	03.05.2010
*Syrista*					
	*Syrista parreyssii* (Spinola, 1843)	ECUUS 280	JF901906	Sivas	26.05.2007
	*Syrista parreyssii*	ECUUS 281	JF901907	Adana	05.06.2003
*Characopygus*					
	*Characopygus* sp.	ECCUS 290	JF901895	İçel	13.04.2009
*Pachycephus*					
	*Pachycephus cruentatus* (Eversmann, 1847)	ECCUS 300	JF901904	Sivas	06.06.2009
	*Pachycephus smyrnensis* J.P.E.F. Stein, 1876	ECCUS 301	JF901908	Edirne	07.05.2010
	*Pachycephus smyrnensis*	ECCUS 302	JF901909	Edirne	07.05.2010
	*Pachycephus smyrnensis*	ECCUS 303	JF901910	Sivas	11.06.2010
	*Pachycephus* sp.	ECCUS 304	JF901905	Sivas	12.06.2010

### DNA extraction, amplification, and sequencing

Alcohol-preserved specimens were allowed to dry on filter paper, and DNA was extracted from left legs of the specimens using the High Pure PCR Template Preparation Kit (Roche Diagnostics, Mannheim, Germany) following the protocol for DNA isolation from mammalian tissue. Each DNA sample was dissolved in 200 µl elution buffer and stored at -20°C. The partial mitochondrial COI gene (750 bp) was amplified by using the conserved COI primers with the following sequence: COI–s1859, 5’ – GGAACIGGATGAACWGTTTAYCCICC – 3’ and COI–a2590 5’ – GCTCCTATTGATARWACATARTGRAAATG – 3’ (Simon et al. 1994). PCR reactions were conducted with 10 µl of extracted DNA in 50 µl reaction mixture. Amplification conditions were as follows: denaturation for 5 min at 94°C, followed by 37 cycles of denaturation at 94°C for 30 s, annealing at 59°C for 45 s, extension at 72°C for 30 s and a 5 min final extension at 72°C. The purification and sequencing of amplification products were performed using a commercial sequencing company (Macrogen Ltd., Seoul, Korea.). Sequencing reactions were carried out in both directions using the same primers as in PCR reactions. The forward and reverse nucleotide sequences were assembled and edited by eye using the CodonCode Aligner v 3.5.6 (CodonCode Corporation) and aligned by using CLUSTAL W version 1.83 (Thompson et al. 1994), using the default parameters of the program. Finally, all the sequences obtained are deposited in GenBank (Table 1).

### Data analysis

Estimates of evolutionary divergence analyses were conducted in MEGA5 (Tamura et al. 2007) using the Kimura 2-parameter model (Kimura 1980) over sequence pairs between genera. The rate variation among sites was modeled with a gamma distribution (shape parameter = 0.87). The presence of substitution saturation was determined with DAMBE version 4.5.18 (Xia and Xie 2001). The genetic distance versus the number of transitions and transversions at first, second and third codon position in all taxa was plotted to examine the saturation at a partial COI gene sequences.

In order to investigate the phylogenetic relationship of Cephinae, phylogenetic trees were constructed using maximum parsimony (MP), maximum likelihood (ML) and Bayesian inference (BI) methods. Nucleotides were used as discrete and unordered characters. The best-fit model of DNA substitution and the parameter estimates used for tree constructions were chosen according to the Akaike Information Criterion (AIC) as implemented in Modeltest version 3.7 (Posada and Crandall 1998). The phylogenetic signal in the data partitions was estimated by maximum likelihood mapping method (Strimmer and von Haeseler 1997) using TREE-PUZZLE version 5.2 program (Schmidt et al. 2002). MP phylogenies were estimated, with characters unordered and equally weighted, under the heuristic search algorithms ‘simple’ and ‘TBR’ using PAUP version 4.0b10 (Swofford 2002). Bootstrap estimates were calculated from 100 replicates under the above search options. This whole procedure was also applied to the data after removal of the third codon position. ML analyses (Felsenstein 1981) were conducted using RAxML-VI-HPC v. 4.0.0 (Stamatakis 2006)f. The AIC results from Modeltest provided the GTR + I + G model as the best-ﬁt for substitution model. BI analysis was performed with the software BEAST v. 1.5.2 (Drummond and Rambaut 2007). The analysis was run with four chains for 5 × 10^7^ generations, sampling from the chain every 5.000 generations. This generated an output of 10^4^ trees. All analyses were performed assuming a Yule process of diversification. In order to confirm that the chains had achieved stationary, we evaluated ‘‘burn-in” plots by plotting log-likelihood scores and tree lengths against generation number using the software Tracer v. 1.5 (Drummond and Rambaut 2007). After determining convergence, we discarded all samples obtained during the first five millions generations as ‘‘burn-in”. The percentage of samples recovering any particular clade in a BI analysis represents that posterior probability of a clade (Huelsenbeck and Ronquist 2001). A majority rule consensus tree (Bayesian tree) was then calculated from the posterior distribution of trees, and the posterior probabilities calculated as the percentage of samples recovering any particular clade. The BI tree built with TREEANNOTATOR, discarding the initial 10% of samples as burn-in [Fig-TREE v. 1.3.1] (Rambaut 2008) was used to visualize the results. For the sake of a better presentation, branches representing individuals belonging to same species were collapsed if the species is recovered as monophyletic (Fig. 4).

## Results

Evaluation of the material collected after publication of Korkmaz et al. (2010) and Calmasur and Ozbek (2010) revealed that there are two additional species of *Cephus* occur in Turkey. The examined material is presented below.

### Cephus parvus

(Dovnar-Zapolskij, 1931)

#### Material examined.

Turkey: Sivas [39°42.71'N, 37°01.30'E] 1300 m, 26.05.2010, 1♀, 17.05.2010, 1♂.

#### Distribution.

Palearctic region.

### Cephus runcator

Konow, 1896

#### Material examined.

Turkey: Edirne [40°39.32'N, 26°17.82'E] 50 m, 07.05.2010, 6♀, 1♂.

#### Distribution.

Turkey, S. E. Europe.

**Figure 1.**
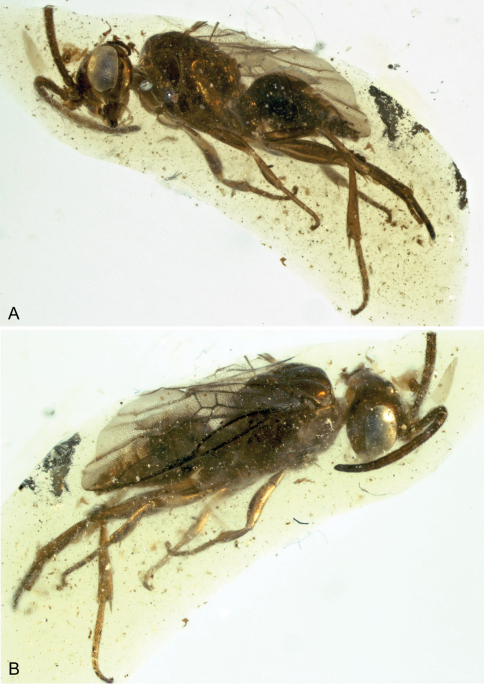
Percentage of nucleotide composition at each codon position.

The complete alignment of the partial mitochondrial COI gene sequences from 68 cephid specimens, including representatives of these two new records, resulted in a fragment containing 658 base pairs, among which 287 nucleotide positions were variable and 223 sites of which were parsimony-informative. The analyzed sequences correspond to a functional mitochondrial gene region because of the presence of singular peaks in each chromatograph and absence of in–del and premature stop codons, and presence of the highest nucleotide substitutions at the third codon position (Avise 1994). The percentages of nucleotide composition at each codon position are variable (Fig. 1). The mean frequency of COI sequences used in the analyses showed a bias of A + T (T 37.0%, C 15.2%, A 33.9% and G 16.0%), which is similar to other reported members of Hymenoptera (Jermiin and Crozier 1994, Dowton and Austin 1995, 1997, Leys et al. 2000, Danforth et al. 2003). The A + T content at the third, second and first codon positions are 90.7%, 59.8%, and 61.9%, respectively. The nucleotide G has lowest (1.0%) and the A highest content (52.8%) at the third codon positions. The distribution of polymorphic sites for all cephid species shows that the majority of substitutions are at synonymous sites. The vast majority of synonymous substitutions are also found at third codon positions with a rate of 87.88% for the Cephinae. The first and the second positions are relatively more conserved in comparison with the third position.

The numbers of base substitutions per site from averaging over all sequence pairs between genera are shown in Table 2. The least diverged genera appears to be *Characopygus* and *Pachycephus* (p= 0.062) and, the most are *Hartigia* and *Syristra* (p= 0.161) also with highest standard error value of 0.017.

**Table 2.** Estimates of evolutionary divergence over sequence pairs between genera. The number of base substitutions per site from averaging over all sequence pairs between groups are shown. Standard error estimates are shown above the diagonal.

Genera	1	2	3	4	5	6	7	8
1. *Calameuta*		0.010	0.011	0.010	0.013	0.012	0.015	0.025
2. *Trachelus*	0.110		0.010	0.009	0.013	0.012	0.014	0.025
3. *Cephus*	0.108	0.119		0.007	0.013	0.010	0.016	0.026
4. *Characopygus*	0.078	0.094	0.069		0.012	0.007	0.012	0.028
5. *Hartigia*	0.136	0.146	0.143	0.114		0.013	0.017	0.029
6. *Pachycephus*	0.116	0.125	0.113	0.062	0.137		0.013	0.029
7. *Syrista*	0.124	0.145	0.156	0.102	0.161	0.125		0.030
8. Outgrup	0.248	0.263	0.256	0.249	0.292	0.278	0.279	

**Figure 2.**
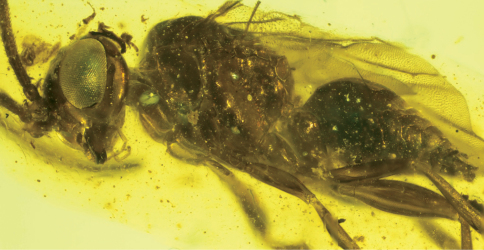
Saturation plots of transversion and transition rates against JC69 distance at **a** first codon position **b** second codon position **c** third codon position, and **d** sum of data.

All three codon positions in the partial COI gene were analyzed for saturation, achieved by plotting the number of observed substitutions against the model TN93 genetic distance estimates. The scattergrams (Fig. 2a–c) showed that transitions and transversions for the first, second and third codons of the partial COI gene increased with the genetic distance, but considerable scattering was also observed. In addition, a similar plot of the third codon transition of the COI gene (Fig. 2d) suggested that saturation of transition occurred between certain pairs of the taxa, which may lead to higher levels of homoplasy (Kumar et al. 2001; Zhang et al. 2008).

**Figure 3.**
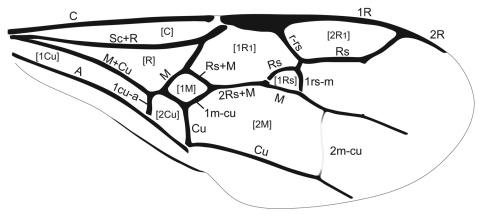
Likelihood mapping analysis of the sequence alignments of COI gene present in the Cephinae. The regions at the corners of the triangles correspond to the three possible tree topologies for a quartet; the lateral regions to partly resolved trees and the central region to unresolved trees. The numbers indicate the percentage of quartets falling in each region.

**Figure 4.**
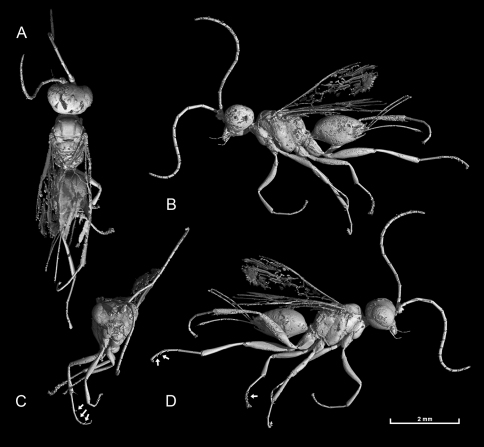
Bayesian interface tree based on the mitochondrial COI gene sequences of the Cephinae. Host plants are indicated in parentheses. Numbers at nodes indicate the posterior values.

Result of likelihood mapping is presented in Fig. 3. High dichotomic phylogenetic signal was detected in the dataset. The percentage of the quartets suggesting a star- or network- like phylogeny is 9.9%, indicating that data are reliable for a dichotomic phylogenetic analysis (Schmidt 2009). For ML analysis GTR+I+G models showed a significantly better fit than the other less complicated models for the COI dataset. Maximum likelihood analyses under the same model of evolution resulted in topologies with lnL = – 5570.6831 in RAxML, which were very close to the BI tree. Bayesian inference under the GTR+I+G model resulted in a topology with mean lnL = – 5347.963. Posterior probability values from the BI were congruent with ML bootstrap support. ML and BI analyses generated a tree with almost the same overall topology (Fig. 4). Equally weighted parsimony analysis of the 287 parsimony-informative characters produced 12 most parsimonious trees with a length of 1065 steps (Homoplasy Index = 0.608, Retention Index = 0.392 and Consistency Index = 0.392). These equally parsimonious solutions were due to differences in terminal branches. However, the branching pattern of bootstrap tree was comb-like and recovered almost no original branches. Considering that this may be due to many synonymous changes in the third codon position, we run an analysis excluding the third codon position from the data. The analysis produced 60 equally parsimonious trees with a length of 200 and the bootstrap application was also resulted with no support to branching pattern of original trees. This may be partly attributed to the nature of data and relatively a short sequence not sufficient to detect phylogenetic signal under parsimony interference. Therefore, we do not present any MP trees here.

## Discussion

Currently, the Cephinae is divided into three tribes based on morphology and feeding habits of larvae. The recovered mitochondrial gene trees substantially conflict with the current taxonomic arrangement, particularly the tribe level. Trees constructed under ML and BI methods supported monophyly of each genus except *Pachycephus* but failed to recover monophyly of any tribes. However, it should be noted that monophyly of most genera were supported by low posterior values (Fig. 4). This is probably due to the strongly biased nucleotide composition and the saturation at the third codon position (Fig. 2). The BI tree suggests that the most basal clade of Cephinae is the genus *Cephus* making the Cephini paraphyletic with respect to rest of Cephini and other tribes. Occurrence of *Syrista* within Pachycephini rather than Hartigiini makes both tribes polyphyletic and paraphyletic respectively (Fig. 4). Otherwise, Pachycephini and Hartigiini appear as sister groups. However, we do not propose a new classification as the present phylogeny is generated from a single gene fragment.

Evolution of phytophagy has occurred many times in insects, and is often accompanied by a significant increase in rates of speciation (Mitter et al. 1988). Phytophagous insects are also notable for their high degree of host-plant specialization; probably over 75% of species feed only on members of one plant family (Bernays and Chapman 1994), and many insect species feed only on a single plant species (Scheffer and Wiegmann 2000). *Syrista* which is considered in the tribe Hartigiini, occurred within Pachycephini clade (see Fig. 4) and this placement is questionable as larvae of *Syristra* feed on *Rosa*. However, if this placement is considered to be true than it suggests a host shift event from Papaveraceae to Rosaceae. Occurrence of *Cephus* at most basal clades also suggests two later shifts from Poaceae to Rosaceae and Papaveraceae (Fig. 4). Considering relationships among genera and species of Cephini inferred from the present phylogenetic hypothesis, several host shift events are also evident. However, we are reluctant for further discussion until a more robust phylogeny become available derived from analyses of several gene sequences of both nuclear and mitochondrial genomes.

## Supplementary Material

XML Treatment for Cephus parvus

XML Treatment for Cephus runcator
